# Targeted p53 activation by saRNA suppresses human bladder cancer cells growth and metastasis

**DOI:** 10.1186/s13046-016-0329-8

**Published:** 2016-03-25

**Authors:** Chenghe Wang, Qiangqiang Ge, Qingsong Zhang, Zhong Chen, Jia Hu, Fan Li, Zhangqun Ye

**Affiliations:** Department of Urology, Tongji Hospital, Tongji Medical College, Huazhong University of Science and Technology, No. 1095 JieFang Avenue, Wuhan, 430030 Hubei China; Department of Urology, Shanghai Jiao Tong University Affiliated Sixth People’s Hospital, No. 600 Yishan Road, Shanghai, 200233 China

**Keywords:** RNA activation, saRNA, Bladder cancer, Proliferation, Metastasis

## Abstract

**Background:**

Previous study showed that dsP53-285 has the capacity to induce tumor suppressor gene p53 expression by targeting promoter in non-human primates’ cells. And it is well known that TP53 gene is frequently mutant or inactivated in human bladder cancer. Hereby, whether this small RNA can activate the expression of wild-type p53 and inhibit human bladder cancer cells remains to be elucidated.

**Methods:**

Oligonucleotide and lentivirus were used to overexpress dsP53-285 and dsControl. Real-time PCR and western blot were used to detect genes’ mRNA and protein expression, respectively. Cell proliferation assay, colony formation, flow cytometry, transwell assay and wound healing assay were performed to determine the effects on bladder cancer cells proliferation and migration/invasion *in vitro*. Animal models were carried out to analyze the effects on cells growth and metastasis *in vivo*.

**Results:**

Transfection of dsP53-285 into human bladder cancer cell lines T24 and EJ readily activate wild-type p53 expression by targeting promoter. Moreover, dsP53-285 exhibited robust capacity to inhibit cells proliferation and colony formation, induce cells G0/G1 arrest, suppress migration and invasion. Besides, the Cyclin-CDK genes (Cyclin D1 and CDK4/6) were down-regulated and the EMT-associated genes (E-cadherin, β-catenin, ZEB1 and Vimentin) were also expressed inversely after dsP53-285 treatment. In addition, dsP53-285 could also significantly suppress the growth of bladder cancer xenografts and metastasis in nude mice. Most importantly, the anti-tumor effects mediated by dsP53-285 were mainly achieved by manipulating wild-type p53 expression.

**Conclusion:**

Our findings indicate that the dsP53-285 can upregulate wild-type p53 expression in human bladder cancer cells through RNA activation, and suppresses cells proliferation and metastasis *in vitro* and *in vivo*.

**Electronic supplementary material:**

The online version of this article (doi:10.1186/s13046-016-0329-8) contains supplementary material, which is available to authorized users.

## Background

Bladder cancer is a major cause of morbidity and mortality worldwide, and with an expected 74,000 newly diagnosed cases and 16,000 deaths in United States in 2015 [1]. Risk factors, such as genetic and molecular abnormalities, chemical or environmental exposures, and chronic irritation, may contribute to the development of bladder cancer [2]. At diagnosis, more than 90 % of bladder cancer patients presents transitional cell carcinoma [3]. Approximately 75 % of newly diagnosed bladder cancer cases appear as non-muscular invasive tumors, they have a high rate of recurrence and progression despite local therapy. The remaining 25 % of newly diagnosed cases are muscle invasive and need either radical surgery or radiotherapy but often still have poor prognosis despite systemic treatment [4, 5].

As an essential tumor suppressor, wild-type p53 is often mutated or inactivated in bladder cancer, especially in muscular invasive tumors [6, 7]. Moreover, independent of tumor grade, stage and lymph-node status, the expressing level of p53 has been implicated as an important predictor of recurrence, progression and survival of patients with bladder cancer [8]. Additionally, alterations of the wild-type p53 play a crucial role in the carcinogenesis of bladder urothelial cancers [9]. Therefore, gain-of-function manipulation of wild-type p53 would be an ideal target for inhibiting bladder cancer cells.

RNA activation (RNAa) is a recently discovered mechanism of gene activation at transcriptional level triggered by small double-stranded RNAs (dsRNAs), and this dsRNAs are termed as small activating RNAs (saRNAs) [10]. What’s more, this novel gene positive regulation mechanism is conserved in at least mammalian cells [11]. A previous study has shown that a candidate dsRNA (dsP53-285) targeting sequence position -285 relative to the transcription start sites (TSS) in the human p53 promoter significantly induced p53 expression in cells of non-human primates [12]. As such, reactivation of p53 through RNAa may offer a promising new therapeutic strategy for bladder cancer.

However, whether dsP53-285 can induce wild-type p53 expression in human bladder cancer cells remains unknown. In the present study, we transfected dsP53-285 into bladder cancer cell lines T24 and EJ for 72 h, and examined the wild-type p53 expression. Our results showed that dsP53-285 had potent ability to inhibit bladder cancer cells proliferation and metastasis by modulating wild-type p53 expression.

## Methods

### dsRNAs and recombinant Lentivirus

All the RNA duplexes used in present study which possesses 2-nucleotide 3’ overhangs were chemically synthesized by RiboBio Co., Ltd. (Guangzhou, China). A small interfering RNA (siP53) was used to silence p53 expression and a dsControl which lacks significant homology to all known human sequences was used as a negative control [10, 13]. Lenti-dsP53-285 and Lenti-dsControl were purchased from GenePharma (Shanghai, China). The sequences of all the custom dsRNAs are listed in Additional file 1: Table S1.

### Cell culture, transfection with dsRNAs and infection with Lentivirus

The human bladder cancer cell lines T24 and EJ (ATCC) were cultured in RPMI 1640 medium (Hyclone, USA) supplemented with 10 % fetal bovine serum (Gibco, USA) in a humidified atmosphere with 5 % CO_2_ at 37 °C. The day before transfection, cells were trypsinized and plated to a new 6-well plate with growth medium at a density of 50-60 % without antibiotics. All dsRNAs were transfected at a final concentration of 50 nM by using Lipofectamine RNAiMax (Invitrogen, USA) according to the manufacturer’s instructions. Besides, dsRNA was replaced by MEM in mock transfection.

The day before infection, 3-5 × 10^3^ cells were seeded in a 96-well plate with 100 μL culture medium, to ensure cells are at a density of 40-60 % in each well when infection. EJ cells were infected with Lenti-dsP53-285 or Lenti-dsControl according to the manufacturer’s protocol with modification. Culture medium was substituted 24 h later. Fluorescence expression was observed at 72-96 h after infection. Then cells were harvested and reseeded into new plates for further experiments.

### RNA isolation and quantitative real-time PCR

Total cellular RNA was extracted from bladder cancer cells by using TRIzol reagent (Invitrogen, USA) according to the manufacturer’s protocol. After quantified by a Nano Drop ND-1000 spectrophotometer, 500 ng RNA was reversely transcribed into cDNA according to the instructions provided by Takara reverse transcription kit (Takara, China). The resulting cDNA was amplified by SYBR Premix Ex Taq II (Takara, China) conducted on the Mx3000P instrument (Stratagene, USA). All the primers included in this study were provided by Invitrogen (Shanghai, China) and listed in Additional file 1: Table S2. The relative expression of target genes’ mRNA was calculated with the 2^-ΔΔCt^ method. GAPDH was used as internal control. All experiments were done in triplicate.

### Protein extraction and Western blotting analysis

All the cells were gathered and total proteins were extracted using RIPA lysis buffer supplemented with protease inhibitor Cocktail (Roche, Switzerland). Protein concentrations were calculated by using BCA protein assay kit (Beyotime, China). Equivalent amounts of protein samples (50 *μ*g) were separated by 10 % sodium dodecyl sulfate polyacrylamide gel electrophoresis (SDS-PAGE) and then transferred to polyvinylidene fluoride (PVDF) membranes. Nonspecific binding was blocked by incubating the PVDF membranes with 5 % bovine serum albumin (BSA) (Sigma-Aldrich, USA) for 2 h at room temperature. The membrane was then incubated with primary antibodies included p53 (1/1000) (Cell Signaling Technology, USA), p21 (1/2000) (Cell Signaling Technology, USA), Cyclin D1 (1/2000) (Affinity, USA), CDK4 (1/1000) (Affinity, USA), CDK6 (1/2000) (Affinity, USA), E-cadherin (1/1000) (BD Biosciences), β-catenin (1/500) (Boster, China), Vimentin (1/500) (Boster, China), ZEB1 (1/1000) (Cell Signaling Technology, USA), GAPDH (1/500) (Boster, China) and α-tubulin (1/500) (Boster, China) at 4 °C overnight. After several washes, the membranes were incubated with corresponding secondary antibody and detected by enhanced chemiluminescence (ECL) assay kit (Millipore, USA).

### Cell proliferation assay

Cell proliferation was detected using the CellTiter 96® AQ_ueous_ One Solution Cell Proliferation Assay kit (Promega, USA) according to the manufacturer’s protocol. Briefly, cells were transfected with indicated dsRNAs in a 6-well plate. The cells were trypsinized and seeded at 1000 cells/well into a new 96-well plate 24 h later. Cell growth was measured at daily interval from the next day to the fifth day. At each time point, 20 μl of CellTiter 96® AQueous One Solution was added to each well and incubated for 2 h at 37 °C. Absorbance was measured on a microplate reader (Bio-Rad, USA) at 490 nm.

### Clonogenic survival assay

T24 and EJ cells were harvested 24 h after transfection of indicated dsRNAs. 1000 cells were reseeded in each new 6-well plate with complete medium for 10 days. The medium was replaced every 3 days to maintain the cells growth. The colonies were then fixed and stained with 0.5 % crystal violet (Sigma, USA) for 30 min at room temperature. The colony formation rate was calculated using the following equation: colony formation rate = number of colonies/number of seeded cells × 100 %.

### Cell cycle analysis by flow cytometry

Cells were harvested 72 h after transfection and fixed with 70 % ethanol at 4 °C overnight. Then the cells were washed and incubated with RNase A (0.1 mg/mL) for 30 min at 37 °C. Cellular DNA was stained with propidium iodide (PI) (0.05 mg/mL) and analyzed on a FACSort flow cytometer (BD Biosciences, USA). All experiments were repeated 3 times and a total of 10,000 events were analyzed for each sample. The data were processed by CELL Quest software (BD Biosciences, USA).

### Wound healing assay

After 72 h transfection, cells were trypsinized and counted. Approximate 5 × 10^5^ cells were reseeded in each well of a new 6-well plate. With incubation overnight, the confluent cells monolayers were scratched with a 10 μL sterile pipette tip. Then the non-adherent cells were washed off with sterilized PBS and serum-free medium was added into the wells. The gap area caused by the scratch was monitored by the inverted microscope (Olympus, Japan). Three random non-overlapping areas in each well were pictured at 0 h, 12 h and 24 h post-scratch. Scratch width between the two linear regions was quantitated for assessing capacity of cells migration.

### Migration and invasion assay

The 24-well Boyden chamber with 8 μm pore size polycarbonate membrane (Corning, USA) was used to analyze the cell motility. For invasion assay, the membrane was pre-coated with matrigel (BD Biosciences, USA) to form a matrix barrier. 2 × 10^4^ cells, transfected with dsRNAs for 72 h, were seeded on the upper chamber with serum-free medium. Medium with 10 % serum was added to the lower chamber as a chemoattractant. The membranes were fixed at 24 h and stained with 0.5 % crystal violet (Sigma, USA). After removal of the non-motile cells at the top of the membranes with cotton swabs, 5 visual fields of 200× magnification of each membrane were randomly selected and counted.

### *In vivo* tumorigenicity assay and experimental lung metastasis model

Equivalent amounts EJ cells (about 5 × 10^6^, 200 μL) infected with Lenti-dsP53-285 or Lenti-dsControl were injected subcutaneously into the right back of male BALB/c-nude mice (Hua Fukang Biological Technology Co., Ltd, Beijing, China) at 4 weeks of age, respectively. Tumor length and width were measured using calipers every 4 days for 28 days. Tumor volume was calculated using the formula: V = length × width^2^ × 0.5. Animals were sacrificed 28 days after injection and tumors were weighed.

For *in vivo* metastasis assay, treated cells (2 × 10^5^) were suspended in 100 μL of PBS and injected intravenously via the tail vein. At 30 days later after injection, the incidence and volume of metastases were estimated by imaging of mice for bioluminescence using the Living Image software (Xenogen, USA). The photon emission level was used to assess the relative tumor burden in the mice lungs. All nude mice were manipulated and cared according to NIH Animal Care and Use Committee guidelines in the Experiment Animal Center of the Tongji medical college of Huazhong University of Science and Technology (Wuhan, China).

### Statistical analysis

All data were presented as the mean ± standard deviation (SD) for three independent experiments. Differences between groups were analyzed by t-tests using SPSS version 13.0 software (SPSS Inc., Chicago, IL, USA). *P*-value < 0.05 was considered to be statistically significant.

## Results

### dsP53-285 activates wild-type p53 expression by targeting promoter

**Fig. 1:**
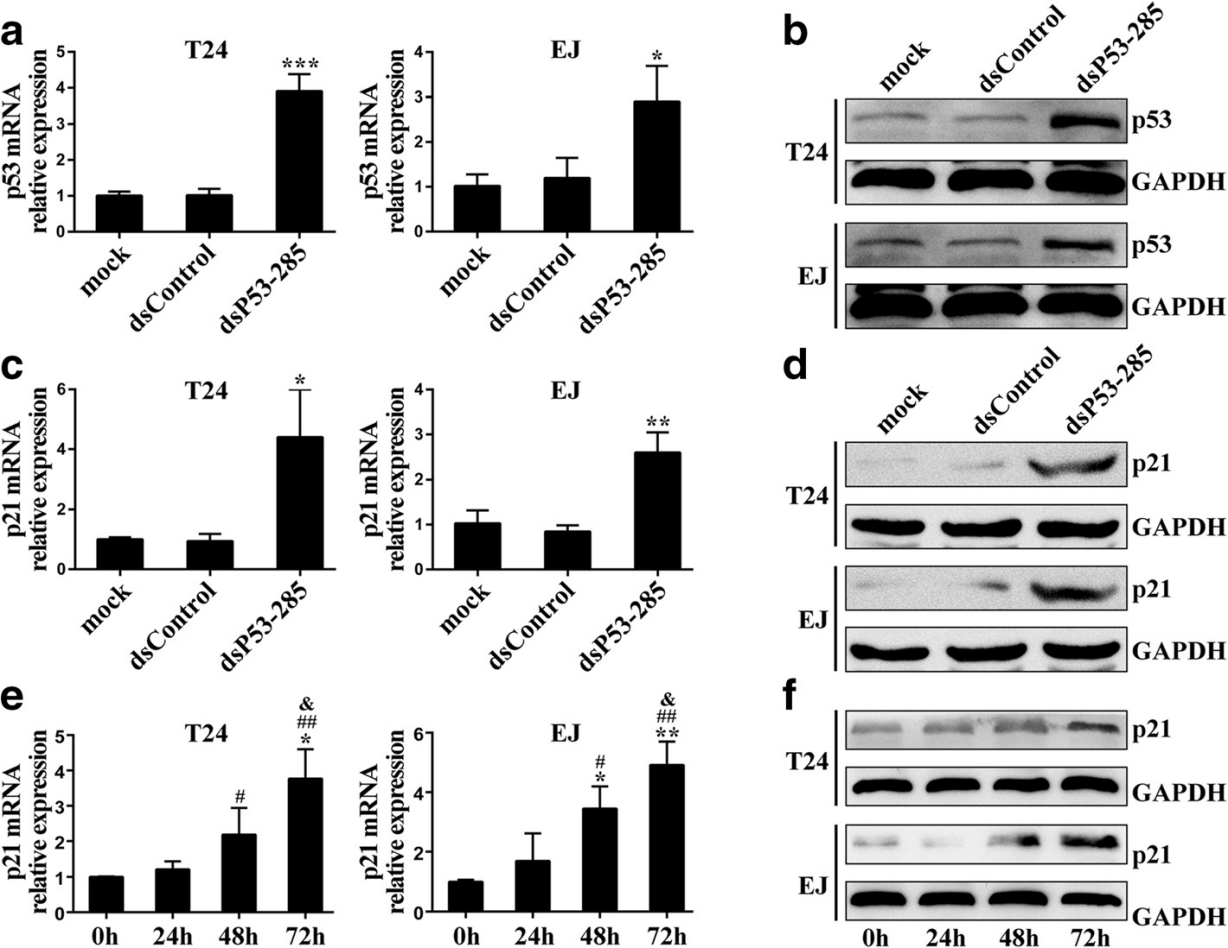
dsP53-285 induces wild-type p53 expression by targeting promoter in human bladder cancer cells. T24 and EJ cells were transfected with 50 nM of the indicated dsRNAs or mock transfection for 72 h. GAPDH levels were detected and served as a loading control. **a** Expression of p53 mRNA levels was assessed by real-time PCR. **b** Expression of p53 protein was detected by Western blot analysis. **c** Expression of p21 mRNA was detected by real-time PCR. **d** Expression of p21 protein was detected by Western blot analysis. ^*^ *P* < 0.05, ^**^ *P* < 0.01 and ^***^ *P* < 0.001 compared to mock and dsControl groups. (**e** and **f**) Time-course of dsP53-285-mediated upregulation of p53 expression. Cells were subjected to real-time PCR or western blot at the indicated time points. ^*^ *P* < 0.01 and ^**^ *P* < 0.001 compared to 0 h; ^#^ *P* < 0.05 and ^##^ *P* < 0.01 compared to 24 h; ^&^ *P* < 0.05 compared to 48 h

Previous study has identified that a specific dsRNA (dsP53-285) can induce p53 expression by targeting sequence position -285 relative to the TSS in the p53 promoter in African green monkey (COS1) and chimpanzee (WES) cells [12]. As human shares almost identical genome sequences with African green monkey and chimpanzee, we speculated that the dsP53-285 may also activate p53 expression in human bladder cancer cells. Hereby, we transfected synthetic dsP53-285 into T24 and EJ cells and analyzed p53 expression 72 h later. Compared with mock and dsControl groups, dsP53-285 caused a significant induction in p53 mRNA (Fig. 1a). This induction was further verified by Immunoblot (Fig. 1b).

It is well known that only wild-type p53 can positively regulate p21 expression [14]. To determine the expression type of p53 in dsP53-285 induced T24 and EJ cells, we further examined the downstream p21 levels. As shown in Fig. 1c, real-time PCR revealed that dsP53-285 profoundly activated p21 levels compared to mock and dsControl treatments. And analysis of p21 induction was further confirmed by western blotting (Fig. 1d). Moreover, time-course experiments revealed that p53 mRNA and protein levels were not induced until 48 h, and continued to increase at 72 h (Fig. 1e and f). Then we silenced wild-type p53 expression by transfecting a small interfering RNA siP53 into both cell lines [13]. The outcomes showed that co-transfection of dsP53-285 and siP53 in the two tested cells markedly abrogated the activating effect of wild-type p53 at both mRNA and protein levels at 72 h (Additional file 2: Figure S3A and S3B). These results suggest that dsP53-285 possesses capacity to induce wild-type p53 expression through RNAa in T24 and EJ cells.

### dsP53-285 inhibits cells proliferation, clonegenesis and induces cell cycle arrest mainly by upregulating wild-type p53 expression

**Fig. 2:**
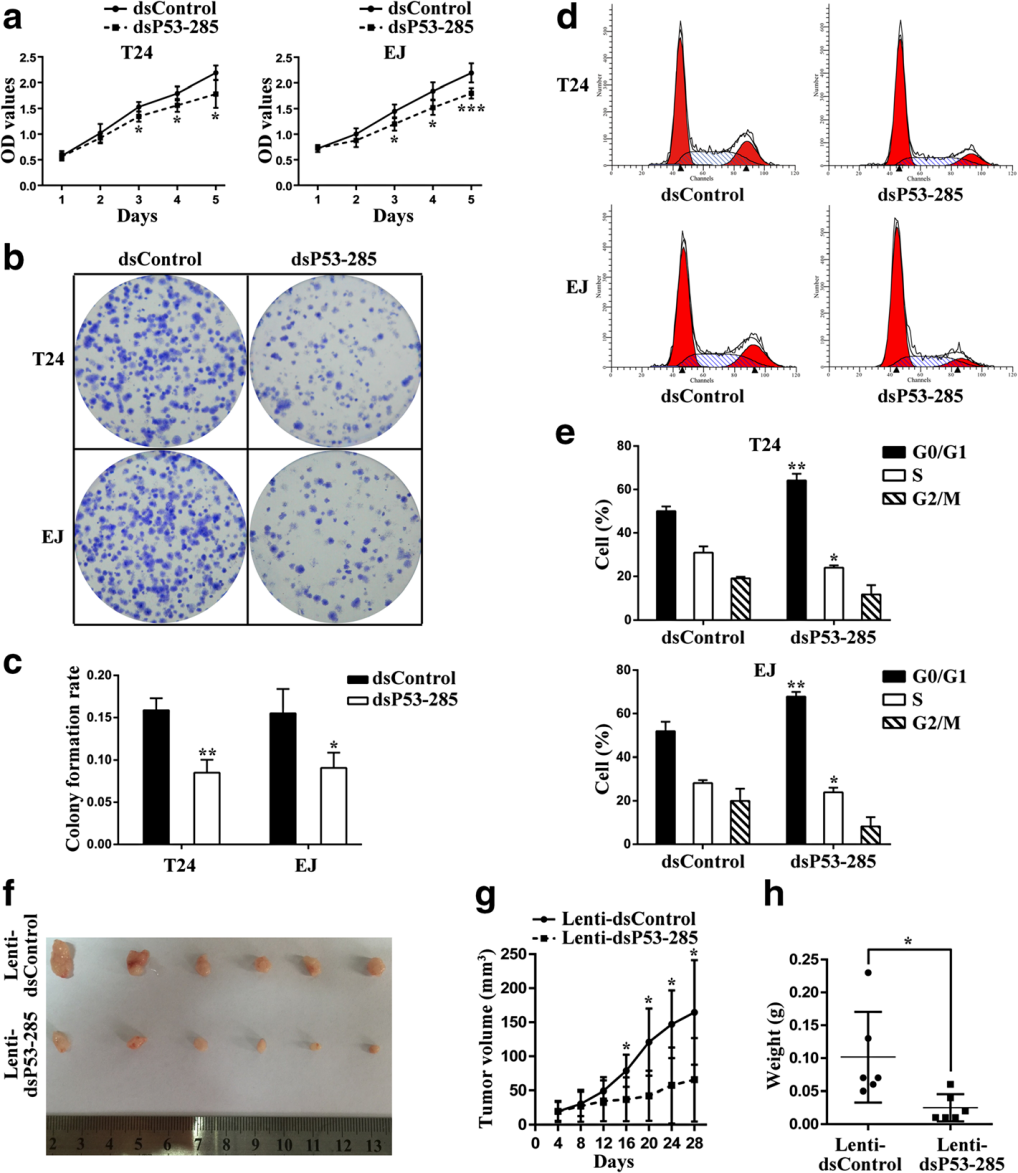
dsP53-285 inhibits cells proliferation, clonegenesis and induces cell cycle arrest. T24 and EJ cells were transfected with 50 nM of the indicated dsRNAs for 72 h. Mock sample was transfected in the absence of dsRNAs. **a** Viable cells were measured from day 1 to 5 following transfection using the CellTiter 96® AQ_ueous_ One Solution Cell Proliferation Assay kit. Results were plotted as OD values. **b** Representative photographs of colony formation assay. **c** Quantification of the cell colonies formation. **d** Representative photographs of cell cycle analysis. **e** Quantification of cell cycle distribution. **f** Photographs of tumors excised 28 days after inoculation of stably transfected cells EJ into nude mice. **g** Mean tumor volume measured by caliper on the indicated days. **h** Tumor weight of each nude mouse at the end of 28 days. ^*^ *P* < 0.05, ^**^ *P* < 0.01 and ^***^ *P* < 0.001 compared to dsControl or Lenti-dsControl group

To investigate the effects of wild-type p53 up-regulation mediated by dsP53-285 on bladder cancer cell lines T24 and EJ, we performed CellTiter 96® AQueous One Solution Cell Proliferation Assay. Compared to dsControl group, both tested cells transfected dsP53-285 exhibited progressive retarded growth from the third day following transfection (Fig. 2a). Then we verified whether depletion of wild-type p53 could affect the inhibitory function of dsP53-285 in T24 and EJ cells. As seen from Additional file 3: Figure S1A, knockout of wild-type p53 evidently attenuated the anti-proliferative effect mediated by dsP53-285 in both cells. Then, the EJ cells stably expressing dsP53-285 or dsControl were used to generate the xenograft model in nude mice. As shown in Fig. 2g, Lenti-dsP53-285 significantly reduced xenograft tumor growth. In addition, the average tumor volume and weight in Lenti-dsP53-285 group was markedly smaller and lighter than the control group at day 28 post injection, respectively (Fig. 2f and h).

In order to evaluate the cells growth mode after wild-type p53 activated by dsP53-285, we carried out the colony formation assay and found that dsP53-285 transfected cells formed colonies significantly fewer in number and smaller in size (Fig. 2b). In addition, the colony formation rates of dsP53-285 transfected cells were remarkably lower than dsControl treatment (Fig. 2c). Furthermore, the colony formation ability of the bladder cancer cells was also restored after co-treatment of siP53 (Additional file 3: Figure S1B and S1C).

Nest we performed flow cytometry to measure cell cycle distribution. In both bladder cancer cell lines, transfection with dsP53-285 led to a significant increase in the G0/G1 population and a corresponding decrease in the S phase population compared with dsControl group (Fig. 2d and e). Additionally, p53 silencing remarkably alleviated G0/G1 cycle arrest mediated by dsP53-285 in both cell lines (Additional file 3: Figure S1D and S1E). These data together implied that dsP53-285 could inhibit bladder cancer cells proliferation, suppress colony formation and induce cell cycle G0/G1 arrest mainly via manipulating wild-type p53 expression.

### dsP53-285 inhibits bladder cancer cells migration and invasion primarily by activating wild-type p53 expression

**Fig. 3:**
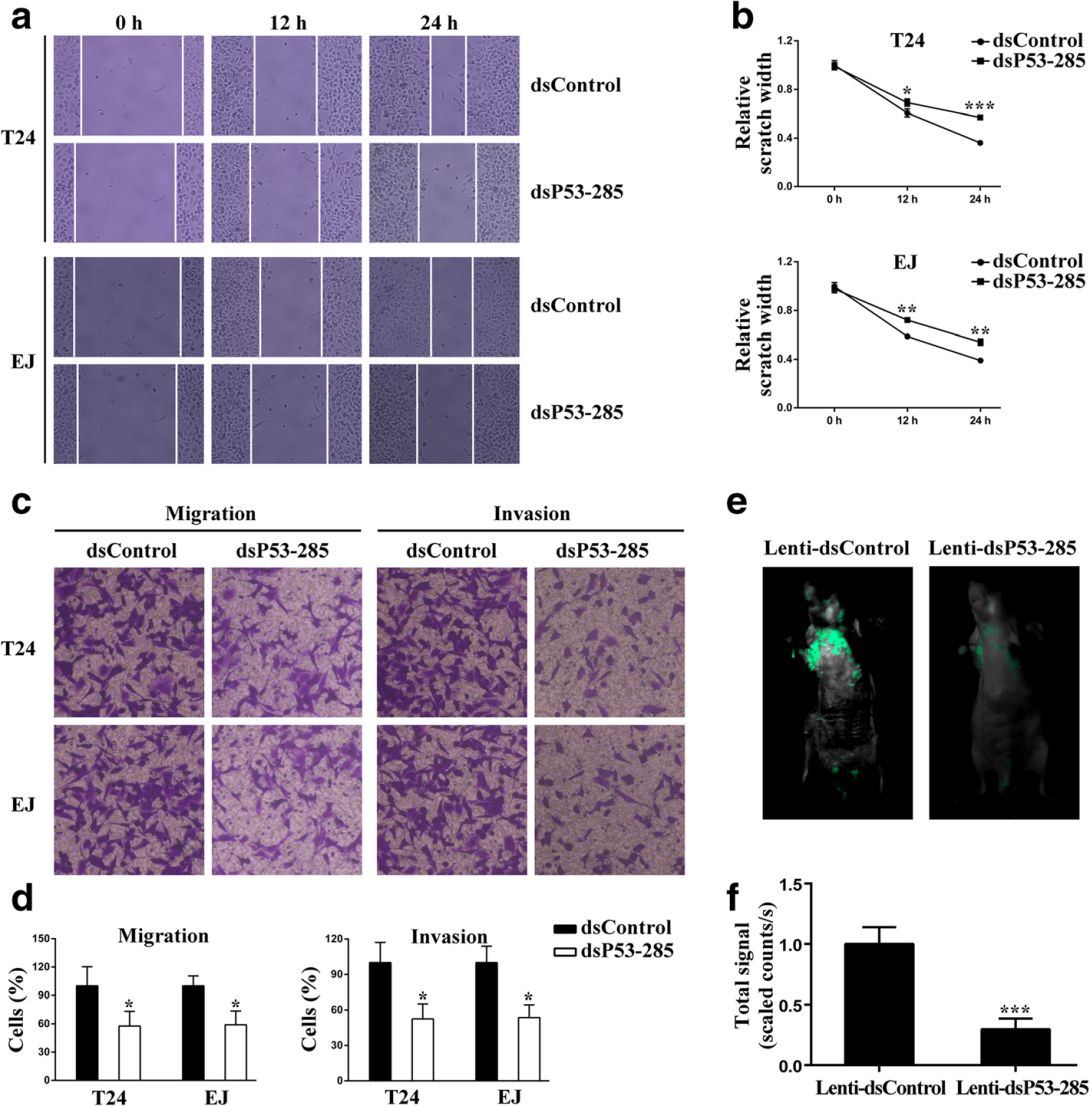
dsP53-285 suppresses bladder cancer cells migration and invasion. T24 and EJ cells were transfected with 50 nM of the indicated dsRNAs for 72 h. **a** Representative wound healing images were pictured at 0, 12 and 24 h. **b** The relative distances between wound edges of bladder cancer cells at 0, 12 and 24 h. **c** Representative photographs of transwell assay (×200). **d** Number of migrated and invaded cells was quantified in 5 random images from each treatment group. Results are plotted as percent (%) relative to dsControl group. **e** Representative bioluminescent images of lungs of nude mice at the 30th days after intravenous injection. **f** Quantification analysis of fluorescence signal from captured bioluminescence images. ^*^ *P* < 0.05, ^*^ *P* < 0.01 and ^***^ *P* < 0.001 compared to dsControl or Lenti-dsControl group

As we know, wild-type p53 suppresses epithelial-to-mesenchymal transition (EMT) which is a process that plays crucial roles in the early stage of metastases, invasiveness and wound healing of bladder cancer [15]. Hereby, we examined the potential roles of dsP53-285 on migration and invasion capacities of bladder cancer cells. At 72 h after transfection, cells were reseeded and then scratches were made 24 h later. Transfection of dsP53-285 led to retarded wound closing compared with dsControl group from 12 h in both T24 and EJ cells (Fig. 3a). Then the wound widths of each group were measured and normalized to corresponding 0 h of dsControl. The relative distances between wound edges of dsP53-285 groups were markedly wider than those of dsControl group in both cell lines from 12 h post-scratch (Fig. 3b). Furthermore, compared to dsP53-285 treatment alone, siP53 co-transfected T24 and EJ cells recovered to close the wound faster within 24 h (Additional file 4: Figure S2A). Further quantitative analysis of wound width indicated relative scratch widths in co-treatment group were significantly less than dsP53-285 transfection groups from 12 h (Additional file 4: Figure S2B).

Subsequently, the transwell assay was conducted to further assess cells migration and invasion capability *in vitro*. T24 and EJ cells transfected with dsP53-285 or dsControl were allowed to migrate through a transwell membrane into complete media. Compared with control, dsP53-285 exerted a potent inhibiting effect on migration of the both cell lines (Fig. 3c and d). Likewise, invasion capability of bladder cancer cells transfected dsP53-285 was detected by Matrigel invasion chamber assay. As expected, dsP53-285 can notably repress their invasion ability compared with dsControl treatment (Fig. 3c and d). In intravenous injection assay, bioluminescence imaging revealed that fluorescence signal in Lenti-dsP53-285 group was significantly weaker than lenti-dsControl group, which mean that less metastasis is formed in lung after dsP53-285 overexpression (Fig. 3e and f). Moreover, depletion of wild-type p53 dramatically restored cells migration in response to dsP53-285 transfection (Additional file 4: Figure S2C and S2D). Consistent with this, cells migrated the membranes pre-coated with matrigel were more in co-transfection group than dsP53-285 group (Additional file 4: Figure S2C and S2D). These results demonstrate that dsP53-285 can suppress the migration and invasion of T24 and EJ cells mainly by regulating wild-type p53 expression.

### dsP53-285 modulates bladder cancer cells cell cycle and EMT associated genes mainly by enhancing wild-type p53

**Fig. 4:**
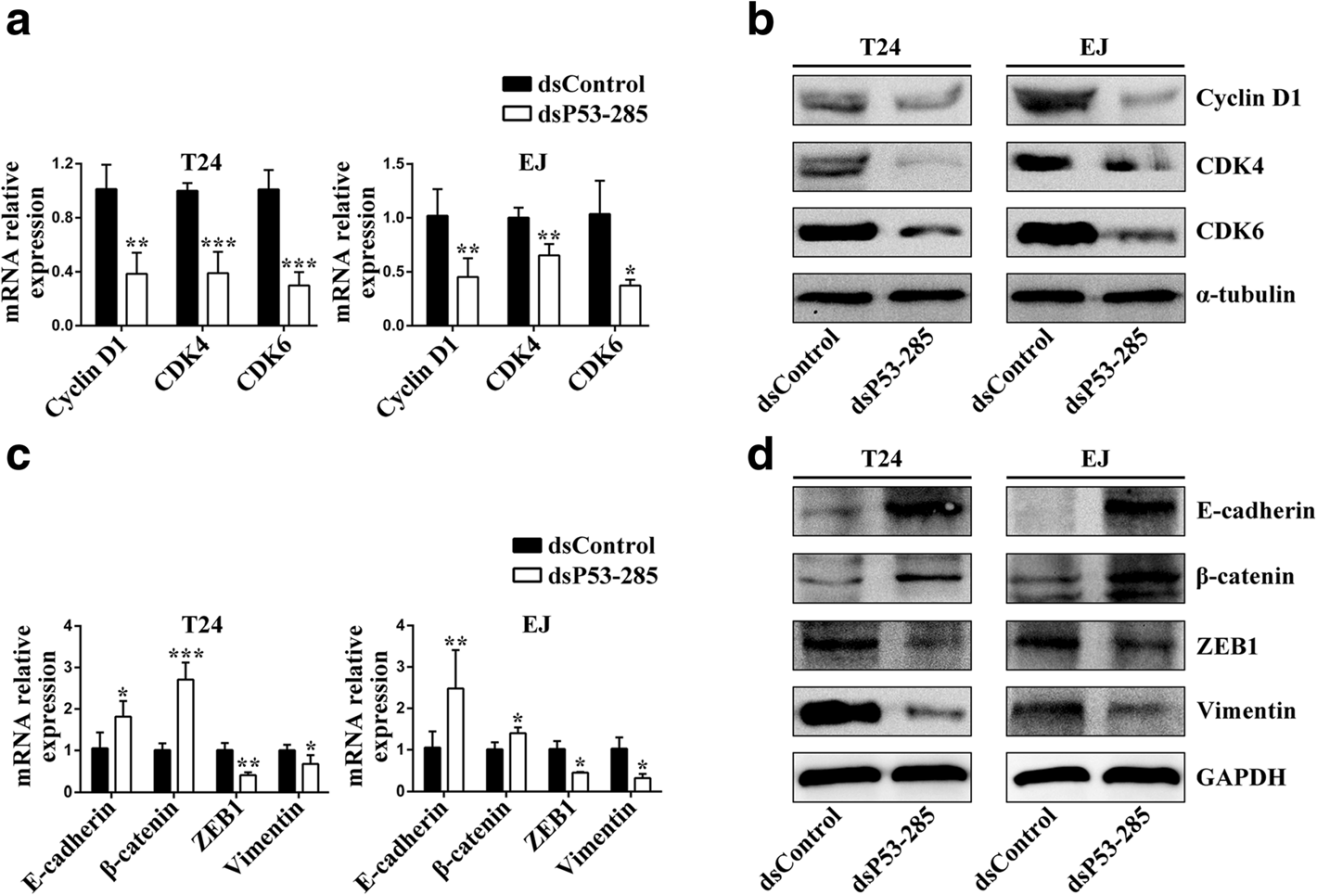
dsP53-285 inhibits Cyclin D1 and CDKs, and reversed EMT-associated genes expression. T24 and EJ cells were transfected with 50 nM of the indicated dsRNAs for 72 h. **a** Expression of Cyclin D1 and CDK4/6 mRNA was detected by real-time PCR. GAPDH served as a loading control. **b** Expression of Cyclin D1 and CDK4/6 protein was detected by Western blot. α-tubulin served as a loading control. **c** Expression of EMT-associated genes mRNA was detected by real-time PCR. GAPDH served as a loading control. **d** Expression of EMT-associated genes protein was detected by Western blot analysis. GAPDH served as a loading control. ^*^ *P* < 0.05, ^**^ *P* < 0.01 and ^***^ *P* < 0.001 compared to dsControl group

We further determined the effects of dsP53-285 transfection on the expression of down-stream genes associated with cell cycle and mitotic checkpoint in bladder cancer cells. As shown in Fig. 4a, transfection of dsP53-285 caused a significant decrease of mRNA levels of Cyclin D1 and CDK4/6 in both tested cell lines. The suppressing effects on protein levels of these genes were further identified by western blotting analysis (Fig. 4b). Moreover, some essential genes related to the EMT process were also detected post dsP53-285 transfection. As seen from Fig. 4c, compared to dsControl group, dsP53-285 significantly up-regulated the mRNA expression of epithelial markers, E-cadherin and β-catenin, and down-regulated mesenchymal markers, ZEB1 and Vimentin in both cell lines, respectively. Immunoblot analysis also revealed a robust increase in E-cadherin and β-catenin, and decrease in ZEB1 and Vimentin protein levels of the two cell lines after dsP53-285 transfection (Fig. 4d).

Next, we blocked wild-type p53 expression and examined the key genes associated with cell cycle. As shown in Figure Additional file 2: Figure S3C, dsP53-285 failed to down-regulated Cyclin D1 and CDK4/6 mRNA levels after siP53 co-transfection. The protein analysis of immunoblot further proved that (Additional file 2: Figure S3D). We next assessed the expression levels of the specific genes mediated EMT process after siP53 transfection. Compared to dsP53-285 group, the mRNA of epithelial markers (E-cadherin and β-catenin) was downregulated, whereas mesenchymal markers (ZEB1 and Vimentin) was upregulated after co-treatment of siP53 for 72 h in both T24 and EJ cells (Additional file 2: Figure S3E). Additionally, immunoblot analysis further confirmed that (Additional file 2: Figure S3F). Taken together, our data strongly imply that dsP53-285 modulates bladder cancer cells cell cycle and EMT associated genes largely depended on modulating wild-type p53 expression.

## Discussion

In the present study, we identified a synthetic dsRNA (dsP53-285) exhibited considerable potency to activate wild-type p53 expression by targeting promoter in human bladder cancer T24 and EJ cells. Moreover, transfection of dsP53-285 induced the cells cycle arrest, impeded growth, migration and invasion. Besides, dsP53-285 could also significantly suppress the growth of bladder cancer xenografts and metastasis in nude mice. Several critical Cyclin-CDK genes (Cyclin D1 and CDK4/6) were down-regulated following transfection. And the EMT-associated genes (E-cadherin, β-catenin, ZEB1 and Vimentin) were also inversely expressed after dsP53-285 treatment. Most importantly, dsP53-285 inhibited bladder cancer cells growth and metastasis *in vitro* and *in vivo* mainly via manipulating wild-type p53 expression.

The activating effect of dsP53-285 molecules on p53 gene by targeting its promoter was initially discovered in African green monkey (COS1) and chimpanzee (WES) cells. Besides, dsP53-285 mediated up-regulation of p53 is conserved in mammalian cells [12]. Therefore, non-human primate disease models may have promising clinical application for validating dsP53-285-based bladder cancer therapeutics.

It is important to point out that the kinetics of RNAa is different from traditional RNA interference. The activation emerges at approximate 48 h and the expressing level of targeted gene continues to increase by 72 h following transfection of specific dsRNA, and lasts for almost 2 weeks [16, 17]. Our finding also showed that p53 expression mediated by dsP53-285 presented a time-course effect. These unique features of RNAa have been attributed to its nuclear nature and consequent epigenetic changes at targeted promoters [10, 11, 16]. Consistent with previous studies, we examined the p53 expression at 72 h post dsP53-285 transfection [18, 19]. What is more, this gene positively regulated phenomenon presents in a dose-dependent manner [10, 20]. So according to other reports [21, 22], we transfected the indicated dsRNAs at a final concentration of 50 nM in our research.

It is disappointed that the exact mechanism of RNAa remains largely unclear [23, 24]. So far, selecting proper dsRNA target sites within specific gene promoter is still a hit-or-miss process [11]. Hence, further studies are needed to improve the target prediction and facilitate to elicit preferable RNAa. In present study, we focus on exploring whether dsP53-285 possessed the ability to stimulate wild-type p53 expression in human bladder cancer cells other than non-human primates’ cells.

The p53 is a well-characterized tumor suppressor, encoded by the TP53 gene located on chromosome 17p13.1 [25, 26]. Analysis of somatic DNA alterations of a recent study showed that nearly half of high-grade muscle-invasive bladder cancers had TP53 mutations and TP53 function was inactivated in 76 % patients [6]. In addition, mutations of TP53 affect one allele, followed by the loss of the wild-type allele, finally disables the function of p53 completely [27, 28]. Thus, reactivation or up-regulation of wild-type p53 would undoubtedly contribute to bladder cancer suppression. Accordingly, our findings strongly argued transfection of dsP53-285 into bladder cancer cells could inhibit their proliferation and metastasis through enhancing wild-type p53 expression.

## Conclusions

Taken together, our study provides evidence that a synthetic dsP53-285 holds potent ability to activate wild-type p53 expression by targeting complementary motifs in promoter region of human bladder cancer T24 and EJ cells. Moreover, dsP53-285 inhibited bladder cancer cells proliferation and metastasis mainly via regulating p53 expression. Nevertheless, further researches are needed to clarify the exact RNAa mechanism and expand the application domain of dsP53-285 in tumor therapeutics.

## Additional files

Additional file 1: Table S1. Sequences for dsRNAs used in present study. Table S2. Sequences for real time quantitative PCR primers used in present study. (DOC 46 kb) Additional file 2: Figure S3. dsP53-285 inhibits Cyclin D1 and CDKs, and reversed EMT-associated genes expression mainly by activating wild-type p53. T24 and EJ cells were transfected with 50 nM of the indicated dsRNAs for 72 h. (**A**) Expression of p53 and p21 mRNA levels was assessed by real-time PCR. GAPDH served as a loading control. (**B**) Expression of p53 and p21 protein was detected by Western blot analysis. GAPDH served as a loading control. (**C**) Expression of Cyclin D1 and CDK4/6 mRNA was detected by real-time PCR. GAPDH served as a loading control. (**D**) Expression of Cyclin D1 and CDK4/6 protein was detected by Western blot. α-tubulin served as a loading control. (**E**) Expression of EMT-associated genes mRNA was detected by real-time PCR. GAPDH served as a loading control. (**F**) Expression of EMT-associated genes protein was detected by Western blot analysis. GAPDH served as a loading control. ^*^ *P* < 0.05, ^**^ *P* < 0.01 and ^***^ *P* < 0.001 compared to dsP53-285 group. (TIF 4114 kb) Additional file 3: Figure S1. dsP53-285 suppresses bladder cancer cells growth largely depended on manipulating wild-type p53 expression. T24 and EJ cells were transfected with 50 nM of the indicated dsRNAs for 72 h. (**A**) Viable cells were measured from day 1 to 5 following transfection using the CellTiter 96® AQ_ueous_ One Solution Cell Proliferation Assay kit. Results were plotted as OD values. (**B**) Representative photographs of colony formation assay. (**C**) Quantification of the cell colonies formation. (**D**) Representative photographs of cell cycle analysis. (**E**) Quantification of cell cycle distribution.^*^ *P* < 0.05 and ^**^ *P* < 0.01 compared to dsP53-285 group. (TIF 7747 kb) Additional file 4: Figure S2. dsP53-285 inhibits bladder cancer cells migration and invasion primarily via enhancing wild-type p53. T24 and EJ cells were transfected with 50 nM of the indicated siP21 and dsRNAs for 72 h. Cell migration and invasion were evaluated after 24 h incubation by transwell assay. (**A**) Representative wound healing images were pictured at 0, 12 and 24 h. (**B**) The relative distances between wound edges of bladder cancer cells at 0, 12 and 24 h. (**C**) Representative photographs of transwell assay (×200). (**D**) Number of migrated and invaded cells were quantified in 5 random images from each treatment group. Results are plotted as percent (%) relative to dsControl group. ^*^ *P* < 0.05 and ^**^ *P* < 0.01 compared to dsP53-285 group. (TIF 20928 kb)

## Abbreviations

BSA: bovine serum albumin
dsRNAs: double-stranded RNAs
ECL: enhanced chemiluminescence
EMT: epithelial-to-mesenchymal transition
PVDF: polyvinylidene fluoride
RNAa: RNA activation
saRNAs: small activating RNAs
SDS-PAGE: sodium dodecyl sulfate polyacrylamide gel electrophoresis
TSS: transcription start sites
