# Supplementary material for: Targeted p53 activation by saRNA suppresses human bladder cancer cells growth and metastasis
Source: J Exp Clin Cancer Res. 2016 Mar 25;35:53. doi: 10.1186/s13046-016-0329-8 (PMC4807596; doi:10.1186/s13046-016-0329-8)
Supplement: Additional file 1: Table S1. — Sequences for dsRNAs used in present study. Table S2. Sequences for real time quantitative PCR primers used in present study. (DOC 46 kb) [file 13046_2016_329_MOESM1_ESM.doc]

**Additional file 1**

**Table S1. Sequences for dsRNAs** used in present study.

| RNAs | Sequences (5’-3’) |
| --- | --- |
| dsControl Sense | ACUACUGAGUGACAGUAGA[dT][dT] |
| dsControl Antisense | UCUACUGUCACUCAGUAGU[dT][dT] |
| dsP53-285 Sense | UUACGGAAAGCCUUCCUAA[dTdT] |
| dsP53-285 Antisense | UUAGGAAGGCUUUCCGUAA[dTdT] |
| siP53 Sense | CUACUUCCUGAAAACAACG[dT][dT] |
| siP53 Antisense | CGUUGUUUUCAGGAAGUAG[dT][dT] |

Table S2. Sequences for real time quantitative PCR primers used in present study.

| Primers | Sequences (5’-3’) |
| --- | --- |
| P53 (F) | CAGCACATGACGGAGGTTGT |
| P53 (R) | TCATCCAAATACTCCACACGC |
| p21 (F) | GCCCAGTGGACAGCGAGCAG |
| p21 (R) | GCCGGCGTTTGGAGTGGTAGA |
| GAPDH (F) | TCCCATCACCATCTTCCA |
| GAPDH (R) | CATCACGCCACAGTTTCC |
| Cyclin D1 (F) | GCTGCGAAGTGGAAACCATC |
| Cyclin D1 (R) | CCTCCTTCTGCACACATTTGAA |
| CDK4 (F) | ATGGCTACCTCTCGATATGAGC |
| CDK4 (R) | CATTGGGGACTCTCACACTCT |
| CDK6 (F) | TCTTCATTCACACCGAGTAGTGC |
| CDK6 (R) | TGAGGTTAGAGCCATCTGGAAA |
| E-cadherin (F) | ACCAGAATAAAGACCAAGTGACCA |
| E-cadherin (R) | AGCAAGAGCAGCAGAATCAGAAT |
| β-catenin (F) | GGAAGATGGGATCAAACCTG |
| β-catenin (R) | TCCGTCTCCGACCTGGAA |
| ZEB1 (F) | ACTCTGATTCTACACCGC |
| ZEB1 (R) | TGTCACATTGATAGGGCTT |
| Vimentin (F) | GATGCGTGAGATGGAAGAGA |
| Vimentin (R) | GGCCATGTTAACATTGAGCA |

F, forward; R, reverse; GAPDH, glyceraldehyde-3-phosphate dehydrogenase. CDK4/6, cyclin-dependent kinase 4/6
