# Supplementary figures and images for: Targeted p53 activation by saRNA suppresses human bladder cancer cells growth and metastasis
Source: J Exp Clin Cancer Res. 2016 Mar 25;35:53. doi: 10.1186/s13046-016-0329-8 (PMC4807596; doi:10.1186/s13046-016-0329-8)

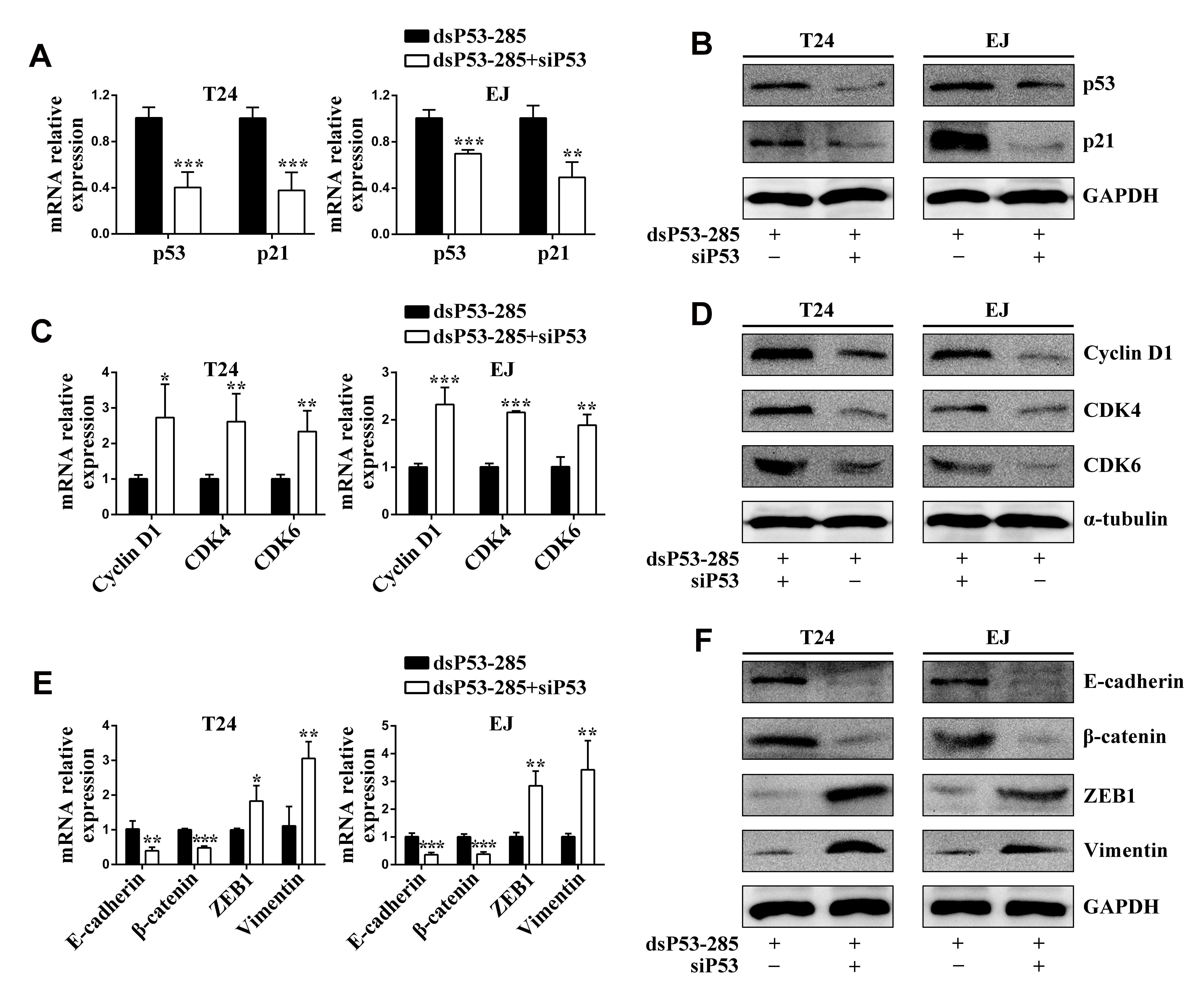

Supplement: Additional file 2: Figure S3. — dsP53-285 inhibits Cyclin D1 and CDKs, and reversed EMT-associated genes expression mainly by activating wild-type p53. T24 and EJ cells were transfected with 50 nM of the indicated dsRNAs for 72 h. (A) Expression of p53 and p21 mRNA levels was assessed by real-time PCR. GAPDH served as a loading control. (B) Expression of p53 and p21 protein was detected by Western blot analysis. GAPDH served as a loading control. (C) Expression of Cyclin D1 and CDK4/6 mRNA was detected by real-time PCR. GAPDH served as a loading control. (D) Expression of Cyclin D1 and CDK4/6 protein was detected by Western blot. α-tubulin served as a loading control. (E) Expression of EMT-associated genes mRNA was detected by real-time PCR. GAPDH served as a loading control. (F) Expression of EMT-associated genes protein was detected by Western blot analysis. GAPDH served as a loading control. * P < 0.05, ** P < 0.01 and *** P < 0.001 compared to dsP53-285 group. (TIF 4114 kb) [file 13046_2016_329_MOESM2_ESM.tif]

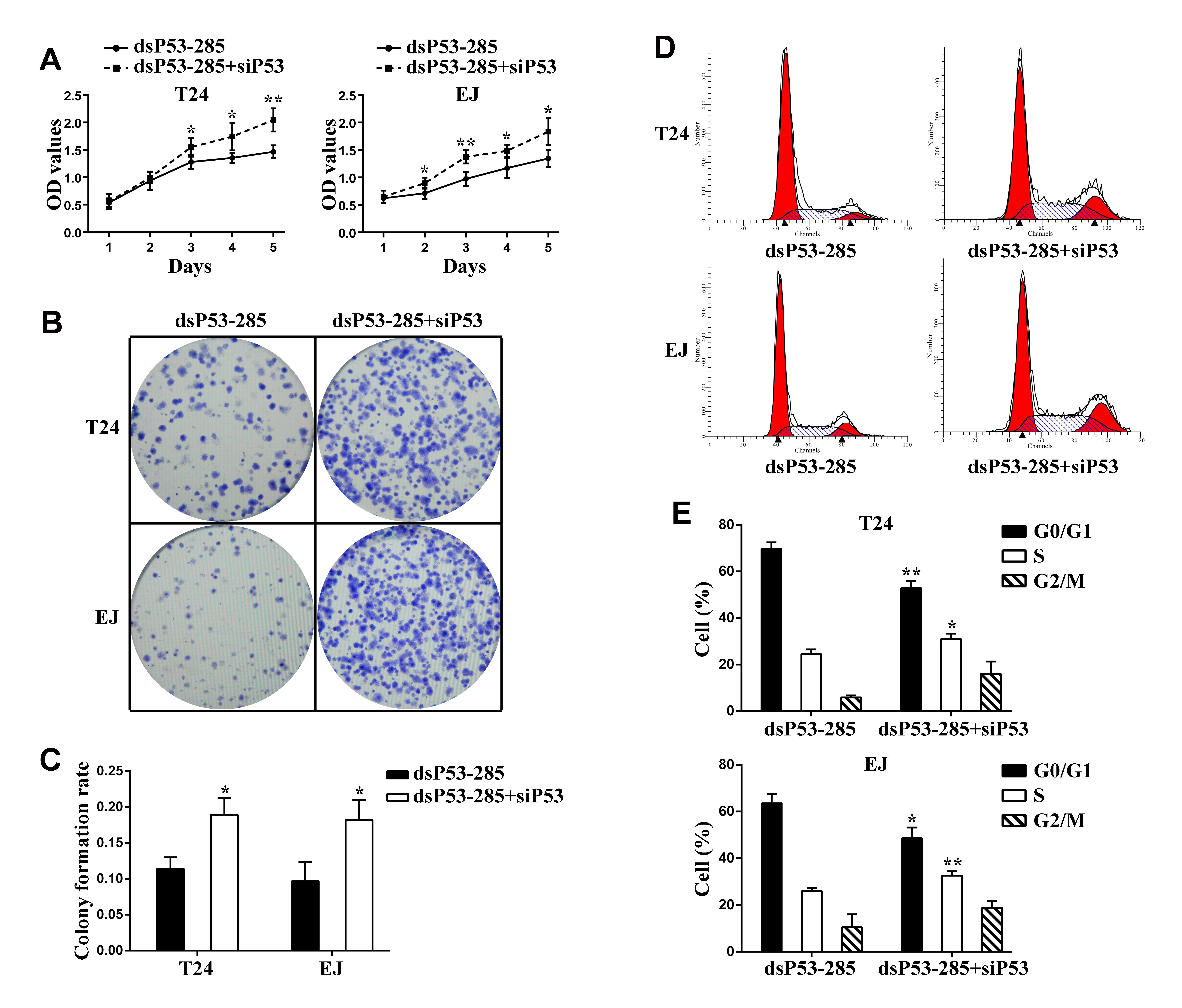

Supplement: Additional file 3: Figure S1. — dsP53-285 suppresses bladder cancer cells growth largely depended on manipulating wild-type p53 expression. T24 and EJ cells were transfected with 50 nM of the indicated dsRNAs for 72 h. (A) Viable cells were measured from day 1 to 5 following transfection using the CellTiter 96® AQueous One Solution Cell Proliferation Assay kit. Results were plotted as OD values. (B) Representative photographs of colony formation assay. (C) Quantification of the cell colonies formation. (D) Representative photographs of cell cycle analysis. (E) Quantification of cell cycle distribution.* P < 0.05 and ** P < 0.01 compared to dsP53-285 group. (TIF 7747 kb) [file 13046_2016_329_MOESM3_ESM.tif]

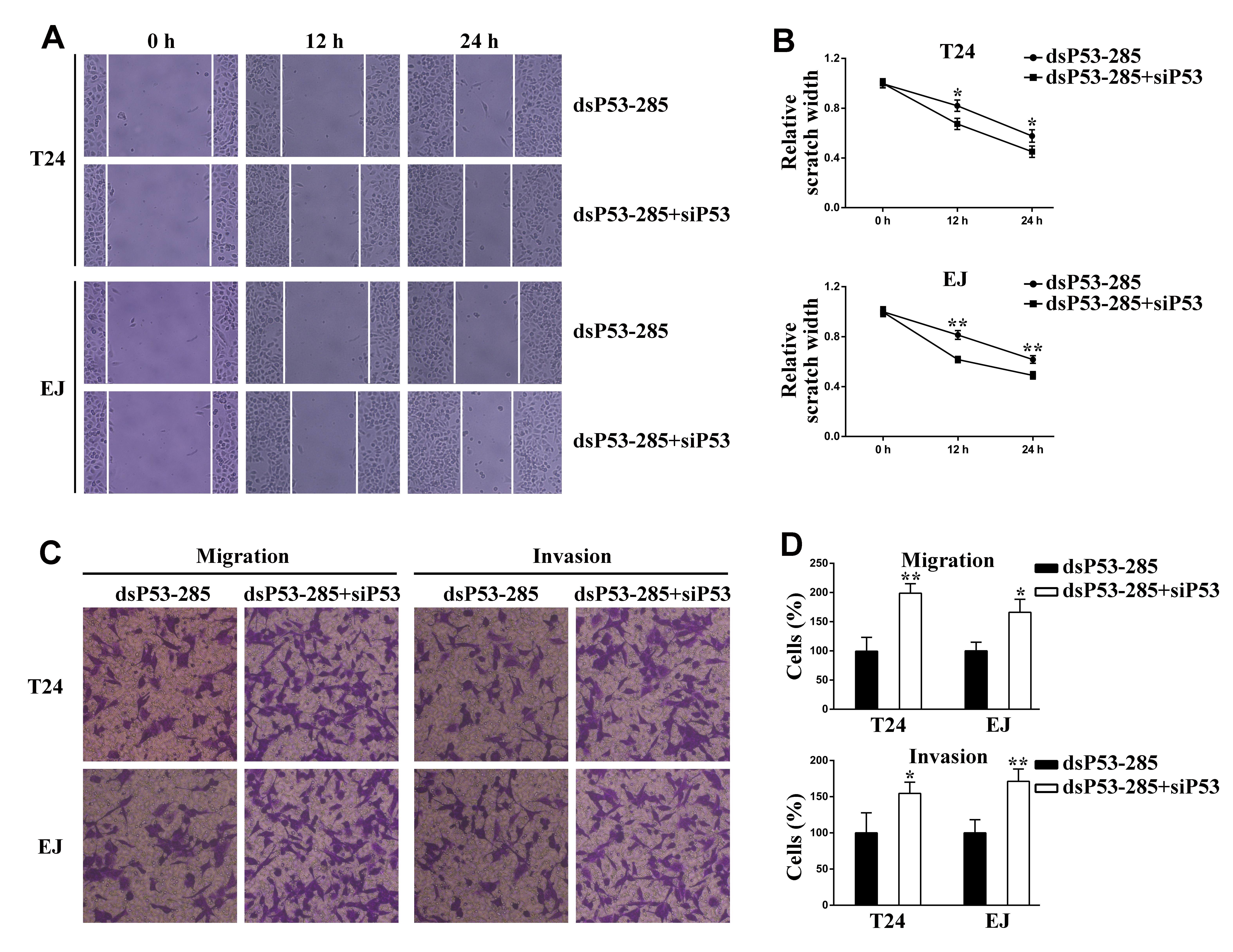

Supplement: Additional file 4: Figure S2. — dsP53-285 inhibits bladder cancer cells migration and invasion primarily via enhancing wild-type p53. T24 and EJ cells were transfected with 50 nM of the indicated siP21 and dsRNAs for 72 h. Cell migration and invasion were evaluated after 24 h incubation by transwell assay. (A) Representative wound healing images were pictured at 0, 12 and 24 h. (B) The relative distances between wound edges of bladder cancer cells at 0, 12 and 24 h. (C) Representative photographs of transwell assay (×200). (D) Number of migrated and invaded cells were quantified in 5 random images from each treatment group. Results are plotted as percent (%) relative to dsControl group. * P < 0.05 and ** P < 0.01 compared to dsP53-285 group. (TIF 20928 kb) [file 13046_2016_329_MOESM4_ESM.tif]
